# The Relationship Between Fear-Avoidance Beliefs, Disability, and Physical Capacity in Patients with Chronic Low Back Pain

**DOI:** 10.31138/mjr.33.3.305

**Published:** 2022-09-30

**Authors:** Shaikh Nabi Bukhsh Nazir, Feliciancus Anthony Pereira, Atta Muhammad, Iram Iqbal Shamsi, Muhammad Uzair Khan

**Affiliations:** 1 Institute of Physical Medical & Rehabilitation, Dow University of Health Sciences, Karachi, Pakistan,; 2 Lecturer, Dewan Institute of Rehabilitation Sciences, Shaheed Benazir Bhutto Dewan University, Karachi, Pakistan,; 3 Lecturer, University Institute of Physical Therapy, University of Lahore, Lahore, Pakistan,; 4 Senior Lecturer, Institute of Physical Medical & Rehabilitation, Dow University of Health Sciences, Karachi, Pakistan,; 5 Post graduate student, Liaquat University of Medical and Health Sciences, Karachi, Pakistan

**Keywords:** low back pain, correlation, pain, psychosocial factors, physical functional performances

## Abstract

**Objective:**

The objective of this study was to document the multi-dimensional profile of patients referring with chronic low back pain (CLBP) and determine the relationship among fear-avoidance belief and disability and physical capacity in the Pakistani population.

**Methods:**

A cross-sectional survey was conducted at the Physiotherapy Department of the Institute of Physical Medicine and Rehabilitation, Dow University of Health Sciences. Consecutive sampling was used among the population of CLBP, and objective measures were fear-avoidance beliefs (FABQ-total) and Roland Morris questionnaire (RMDQ) and pain. Each participant performed a physical capacity test, which included a 6-minute walk test (6MWT), abdominal muscular endurance (AME), hand grip strength (HGS), and Functional reach test (FRT).

**Result:**

Of the 136 participants, 70(51.5%) were males. The mean and SD of the tests performed were: 6MWT (487.97±51.46), AME (9.31±4.68), FRT (35.14±2.79), HGS (33.31±14.55), VAS (5.51±1.27), RMDQ (18.25±2.17), FABQ-total (48.18±5.31). Multivariate regression revealed that symptom duration, FRT, AME, HGS, VAS, RMDQ were all found to be associated with fear-avoidance belief, accounting for 60% (adjusted R^2^ = 0.60) of variability. Likewise, only 6-MWT, FRT, FABQ-total were the significant predictors of disability (adjusted R^2^=0.282).

**Conclusion:**

It has been demonstrated that sub-maximal exercise capacity, strength, endurance, pain and flexibility are the contributing factors for the change in disability and fear-avoidance beliefs among the individuals with CLBP. The findings of this study could have inference for increasing productivity both at individual and organizational levels by formulating effective strategies to manage CLBP.

## INTRODUCTION

Pain that lasts longer than 12 weeks, despite treatment of initial injury, is termed as CLBP.^1^ Individuals who do not seem to recover in this time frame undergo a slower recovery, which burdens the healthcare system, and is a reason of absenteeism from work in 38% of cases reported in Pakistan with CLBP.^2,3^

Treatment options of low back pain are not only based on the duration of symptoms but also the cause, radicular symptoms, and any potential anatomical abnormalities.^4^ Many different factors play a role in the disability regarded with CLBP. Prognostic psychosocial factors need to be assessed in patients with CLBP. Fear-avoidance belief is one factor that is commonly evaluated.^5^

According to the diathesis stress model, distinct characteristics present before the onset of CLBP are stimulated in this stressful period; this can lead to eluding and incapacitating attitudes.^6^ Age and gender are considered risk factors. With regard to gender, there is a greater frequency of low back pain in females as compared to males.^7^

The fear-avoidance model suggests that patients with LBP tend to avoid those activities that are anticipated to cause pain. In long run, this avoidance behaviour results in disuse and deconditioning, thus causing impaired performance in physical tasks.^8^

Physical capacity at the level of the person embodies concepts such as strength, flexibility, endurance, and balance - acts as a pathway that facilitates the individual ability to perform an activity. Reduced capacity due to deconditioning contributes to activity restrictions. Physical capacity in previous research was confined to VO_2_ max, but other studies varied regarding the outcome measure used were trunk muscle static and dynamic strength, walking velocity, and flexibility.^9–11)^ Higher muscle strength, aerobic capacity, endurance, and flexibility as physical capacity variables contribute to greater improvement in the severity of psychological state and cognitive ability.^12,13^

Although different studies have suggested a more or less positive relationship of physical capacity with fear of injury and disability, there is limited evidence to support this conjecture, thus requiring further investigation.^10,14,15^ Their findings might have been more conclusive if the studies included reliable objective measures of fear of injury, both genders, and the onset of symptom duration.^10,14,15^

Therefore, the present study was planned to determine the relationship between disability and physical capacity and fear-avoidance beliefs of those attending the physiotherapy outpatient department due to CLBP, while also considering the different profiles of patients.

## METHODOLOGY

A cross-sectional study was carried out from June 2019 to January 2020, at the Institute of Physical Medicine and Rehabilitation, Dow University of Health Sciences. Following the approval from the institutional review board (IRB-UOL-FAHS/732/2020), sample size of 114 was calculated using Open-Epi 3.0 with a confidence interval CI of 95% and 5% precision. Prevalence was estimated from a previous study conducted on the country survey of Germany where 92% of all participants had low back pain.^16^ Consecutive sampling technique was used for patient selection. Inclusion criteria included: individuals with low back pain of either gender lasting longer than 3 months. Those who had undergone recently laparoscopic or spinal surgery, those with the presence of musculoskeletal disorders or pregnancy or neurological problems such as stroke, those who had low back pain with underlying specific cause were excluded. Those who reported pain during the testing procedure of exercise capacity were also excluded. A written informed consent form was provided before enrolment to ask for their consent for participation in the study. After obtaining informed consent, a self-administered questionnaire was given to them. Participants who could read were given forms to self-response; those unable to do so filled their forms with the help of their caretakers. The survey collected information sheet consisted of a close-ended questionnaire with socio-demographic factors including the Fear-Avoidance Beliefs Questionnaire (FABQ), Roland Morris Questionnaire (RMDQ), Visual Analogue Scale, and Physical capacity test. 6-minute walk test (6-MWT), abdominal muscle endurance (AME), Functional reach test (FRT) and Hand Grip Strength (HGS) were recorded as a measure of physical capacity (PC) variables.

RMDQ is a self-reported questionnaire used as an indicator of level of disability in individuals with low back pain. It comprised of 24 questions with a total score of 24, which had both an Urdu and an English version. The score of up to 15 indicates low-level disability and greater than 15 is considered as a high level of disability. It has a reliability of 0.91.^17^

FABQ is another self-reported 16-item questionnaire consisted of two subscales, FABQ-physical activity (FABQ-P) and FABQ-work (FABQ-W); FABQ-P used to assess belief and attitude towards physical activities (5 items, range 0–30), FABQ-W is focused specifically to assess the attitude towards the work (11 items, range 0–66). A low score on both subscales shows weak fear-avoidance beliefs.^18^

VAS is a one-dimensional indicator of pain severity assessed in either vertical or horizontal orientation line of 1–100 mm. VAS is suggested as: no pain (0–4 mm), mild (5–44 mm), moderate (45–74 mm), and severe (75–100 mm). Its ICC value is 0.97.^19^

6MWT is a sub-maximal exercise performance test to assess the aerobic capacity of an individual by measuring the distance travelled in 6 minutes. ICC=0.87^20^

HGS was checked through a dynamometer; subjects dominant 2^nd^–5^th^ mid-phalanx facing the handle for 3 seconds.^15^ AME was measured on basis of complete repetitions of sit-ups in 30 seconds.^15^ FRT was also carried out to determine the flexibility by measuring a distance the subject can reach forward while in a fixed sitting position.^15^ The distance was measured through the tape.

Multivariable linear regression was applied in identification of predictors of PC in RMDQ and FABQ in individuals with CLBP. The scores obtained from FABQ and disability were analysed and entered separately as dependent variables. The variables used were adjusted with age, gender, Body Mass Index, and the onset of symptom duration. P<0.05 was regarded as significant, and all statistical tests were performed using SPSS 23.0 (IBM, Armonk, NY, USA).

## RESULTS

In total, 136 (150) patients met the inclusion criteria; the sample consisted of an average age of 40.16±10.61 with males in majority 70 (51.5%) **Table 1**. Most of the participants had symptom duration from 6 months to 1 year 55 (40.4%). The mean and SD of the tests performed were: 6MWT (487.97±51.46 m), AME (9.31±4.68), FRT (35.14±2.79 mm), HGS (33.31±14.55 kg.f), VAS (5.51±1.27), RMDQ (18.25±2.17), FABQPA (16.27±1.40), FABQ-W(19.02±2.77), FABQ-total (48.18±5.31).

**Table 1.** Clinical and demographics characteristics of patients with CLBP.

**Variables**	**N=136**
**Gender n (%)**	
Male	70(51.5%)
Female	66(48.5%)
**Onset of symptom duration n (%)**	
3month–6month	51(37.5%)
6month–1year	55(40.4%)
1year–2year	13(9.6%)
>2year	17(12.5%)
**Professional status n (%)**	
Unable to work due to CLBP	56(41.2%)
Active	80(58.8%)
BMI (kg\m2), mean (SD)	28.3(3.84)
Age (years), mean (SD)	40.1(10.61)

**Table 2** lists the correlation between 6MWT, AME, FRT, HGS, VAS, RMDQ and FABQ-tot. 6MWT (r=−0.477*, P **<** 0.01), FRT (r=0.326, P **<** 0.01), HGS (r=0.425, P **<** 0.01) and VAS (r=0.239, P **<** 0.01) showed significant correlation with FABQ-T. 6MWT (r=−0.684, P **<** 0.01), AME (r=−0.325, P **<** 0.01) and FRT (r=−0.374, P **<** 0.01) showed significantly negative correlation with RMDQ. **Table 3** lists the Multivariable linear regression determinants of FABQ-Total and RMDQ. In model 1 (FABQ-Total), the value of R-squared is 0.600, which means that there is 60% of the variation that is explained by this general linear model. Symptom duration, AME, FRT, HGS, VAS, and RMDQ were the statistically positive predictors of the dependent FABQ-Total variable. As the onset symptom duration increased by 10 units, there is the risk of fear-avoidance belief increases by 2.54 points; as the Abdominal muscular endurance increased by 10 units, there is the risk of fear-avoidance belief increases by 3.44 points. As the functional reach test increased by 10 units, there is the risk of fear-avoidance belief increases by 2.44 points; as the hand grip strength increased by 10 units, there is the risk of fear-avoidance belief increases by 4.99 points. As the visual analogue scale increased by 10 units, there is the risk of fear-avoidance belief increases by 1.98 points; as the disability scale increased by 10 units, there is the risk of fear-avoidance belief increases by 2.24 points. In model 2 (RMDQ), the value of R-squared is 0.282, which means that there is 28.2% of the variation that is explained by this general linear model. 6MWT, FRT, and VAS were the statistically negative predictors of the dependent RMDQ variable. For every 10 unit decrease in 6MWT, there is 3.84 points increase in the RMDQ. As the functional reach test decreased by 10 units, there is the risk of RMDQ increases by 2.12 points; only one outcome measure revealed every 10 unit increase of fear-avoidance belief could lead to an increase in 4.03 points of disability.

**Table 2.** Correlation among 6MWT, AME, FRT, HGS, VAS, RMDQ, and FABQ-tot (N=136).

**Variables**	**6MWT**	**AME**	**FRT**	**HGS**	**VAS**	**RMDQ**	**FABQ-tot**
6MWT	1						
AME	0.725 **	1					
FRT	0.447 **	0.418 **	1				
HGS	0.246 **	0.085	0.373 **	1			
VAS	0.041	−0.337 **	−0.086	0.182 *	1		
RMDQ	−0.684 **	−0.325 **	−0.374 **	−0.051	−0.241	1	
FABQ-tot	−0. 477 **	0.021	0.326 **	0.425 **	0.239 **	0.146	1

* Correlation is significant at the 0.05 level (2-tailed);

** Correlation is significant at the 0.01 level (2-tailed); Fear-Avoidance Beliefs Questionnaire: FABQ-tot; Roland Morris Questionnaire: RMDQ; Visual Analogue Scale: VAS; 6-minute walk test: 6MWT; abdominal muscle endurance: AME; Functional reach test: FRT; Hand Grip Strength: HGS.

**Table 3.** Multivariable linear regression analysis of determinants of FABQ-total and RMDQ.

**Model**		**Unstandardised Coefficients**		**Standardised Coefficients Beta**	**P-value**	**95% CI**

		**B**	**Std. Error**			
1	(Constant)	5.763	8.596		0.504	(−11.250, 22.777)
	Gender	0.566	0.626	0.053	0.368	(−0.674, 1.805)
	Age	0.038	0.028	0.076	0.180	(−0.018, 0.093)
	BMI	0.067	0.079	0.048	0.399	(−0.089, 0.223)
	onset of symptom duration	1.367	0.393	0.254	0.001*	(0.590, 2.144)
	6-minute walk test	−0.010	0.010	−0.101	0.291	(0.030, 0.009)
	Abdominal muscular endurance	0.391	0.101	0.344	0.000*	(0.192, 0.590)
	Functional reach test	0.463	0.142	0.244	0.001*	(0.182, 0.745)
	Hand Grip Strength	0.182	0.028	0.499	0.000*	(0.127, 0.237)
	Visual Analogue Scale	0.826	0.274	0.198	0.003*	(0.285, 1.368)
	Rolland Morris Disability	0.549	0.156	0.224	0.001*	(0.241, 0.857)

2	(Constant)	25.398	4.135		0.000	(17.213, 33.583)
	Gender	0.022	0.344	0.005	0.950	(−0.660, 0.703)
	Age	0.010	0.015	0.048	0.526	(−0.021, 0.040)
	BMI	0.018	0.043	0.033	0.671	(−0.067, 0.104)
	onset of symptom duration	0.020	0.225	0.009	0.931	(−0.426, 0.465)
	6-minute walk test	−0.016	0.005	−0.384	0.002*	(−0.027, −0.006)
	Abdominal muscular endurance	−0.010	0.058	−0.022	0.859	(−0.126, 0.105)
	Functional reach test	−0.165	0.080	−0.212	0.041*	(−0.322, −0.007)
	Hand Grip Strength	−0.018	0.018	−.0121	0.306	(−0.053, 0.017)
	Visual Analogue Scale	−0.303	0.153	−0.177	0.050	(−0.606, −0.001)
	FABQ-total	0.165	0.047	0.403	0.001*	(0.072, 0.257)

FABQ model 1; ANOVA P<0.001; adjusted R2 0.600; ^*^Statistical significance at P < 0.05.

RMDQ model 2; ANOVA P<0.001; adjusted R2 0.282; *Statistical significance at P < 0.05.

## DISCUSSION

We found that FABQ-T is strongly associated with VAS, symptom duration, AME, disability, and FRT. This important finding reflects that disability affects not only the performance capacity but also how individuals interpret their pain. This result corroborates with previous work of Zale et al. in which authors determined that strong fear-avoidance beliefs were indicative of occupation-related disability among individuals with sub-acute low back pain. Findings of the systematic review propose that pain-related anxiety may predict disability evaluated during initial course of transformation from sub-acute to chronic pain.^21^

In the current study, FABQ total score was 48.18±5.31. A possible explanation for this finding may be attributed to the degree of disability and pain as measured by the RMDQ 18.25±2.17 and VAS 5.51±1.27. Present study findings contrast the finding of the previous study, which might be due to higher mean age and inclusion of LBP patients with radiating pain and history of the accident, in the majority cases.^22^

The average score of FABQ-PA in this study was 16.27±1.4 which was not parallel with Salama et al.^23^ Their study reported the mean score of 21.2 ± 5.8 and the findings of the study of Guclu et al. was 14.57 ± 6.25.24) Our study had a larger sample size of 136 with the male majority of 70 (51.5%) which might be the reason for variation in the degree of physical activity across these studies.

In the present study, FABQ-W was 19.02±2.77, which was not consistent with findings of Salama et al, who documented a mean score of 30.5 ± 11.4.^23^ The present finding is not in line with the result of Guclu et al who reported a score of 15.19 ± 9.69.^24^ The plausible reason for this inconsistency might be the subjects, who were away from work due to illness.

In the present study, RMDQ mean score was 18.25±2.17. The finding of this study is in contrast with the previous study demonstrated a mean score of RMDQ 10.7**±**4.4, which might be a result of a small sample size of 55. Out of 55 subjects with LBP, 65.5% had chronic pain in Chung et al study.^22^ Apart from flexibility, RMDQ was also significantly associated with sub-maximal exercise capacity and fear of injury in this study. The present study finding is further supported by the research of Verbunt et al., who obtained a mean RDQ score of 11.45: females felt distinctly more disabled than males and reported a significant association between fear and disability. However, they were not able to confirm the physical de-conditioning with fear of injury using the VO_2_ max.^14)^ The current study purported the link between sub-maximal exercise (aerobic capacity) with fear of injury (r=−0.477*, P=0.000).

There was significant correlation between FABQ-T with FRT (r=0.326), HGS (r=0.425) and VAS (r=0.239). The previous study, which was conducted on association of fear-avoidance beliefs with pain and disability in Mexicans with CLBP, identified a positive association among functional disability (r=0.603, P<0.001), recorded on the Roland–Morris scale, and pain (*r* = 0.234, *p* = 0.03) with high scores on the FABQ as a dependent variable. However, FABQ demonstrated significant differences in gender, with greater scores in males as compared with females.^25^ Conversely, another study found an inverse relationship between disability and depression and anxiety levels. The same association was present between physical function and anxiety and depression. Regression model testing revealed that there were significant effects of independent variables on physical function (F=16.722; p=0.000). Physical functionality at a rate of 0.251 (R2=0.251) was attributed to pain severity and fear avoidance.^24)^

On further exploration of the amount of relationship of the fear-avoidance model, found a positive relationship with symptom duration and hand grip strength checked on the side with pain. However, previous research conducted on the exploration of correlation reported a negative relationship between two variables because the sample size consisted of males with a lower mean age.^15^

There are at least two potential limitations concerning the results of this study. The first limitation concern is selection bias, as it was a single-centre study carried at the outpatient physiotherapy department. Another possible limitation was that the study had not enrolled the participants who were taking medications. Despite these limitations, the present study has enhanced our understanding on the relationship of fear-avoidance belief and disability and physical capacity of individuals with CLBP. We expect that the present research will inspire additional investigation in this important area.

## CONCLUSION

Changes in disability and fear-avoidance beliefs may be an important mediating variable for sub-maximal exercise capacity, strength, endurance, and flexibility in individuals with CLBP. The findings of this research may have implications for increasing productivity at the individual and organizational levels through the formulation of effective strategies for the management of CLBP.

## ABBREVIATIONS

6MWT: 6-minute walk test
AME: Abdominal muscular endurance
CLBP: Chronic low back pain
FABQ-total: fear-avoidance beliefs
FRT: Functional reach test
HGS: Hand grip strength
RMDQ: Roland Morris Disability questionnaire
VAS: Visual Analogue Scale
